# Ultrafast Spin Relaxation of Charge Carriers in Strongly Quantum Confined Methylammonium Lead Bromide Perovskite Magic-Sized Clusters

**DOI:** 10.1021/acsphyschemau.4c00051

**Published:** 2024-09-16

**Authors:** David C. Zeitz, Vivien L. Cherrette, Sarah A. Creech, Yan Li, Yuan Ping, Jin Z. Zhang

**Affiliations:** † Department of Chemistry and Biochemistry, University of California, Santa Cruz, California 95064, United States; ‡ School of Materials Science and Engineering, University of Science and Technology Beijing, Beijing 100083, People’s Republic of China; § Department of Materials Science and Engineering, University of Wisconsin, Madison, Wisconsin 53706, United States

**Keywords:** Spin dynamics, nanoclusters, magic-sized clusters, metal halide perovskite, ultrafast

## Abstract

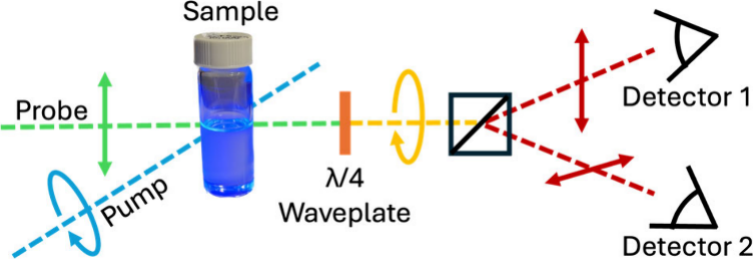

Spin relaxation of charge carriers in strongly quantum confined perovskite magic-sized clusters has been probed, for the first time, by using polarization-controlled femtosecond transient absorption (fs-TA) spectroscopy. Fs-TA measurements with a circularly polarized pump and probe allowed for the determination of the exciton spin relaxation lifetime (∼1.5 ps) at room temperature based on the dynamics of a photoinduced absorption (PIA) feature peaked at 458 nm. This spin lifetime is shorter than that of perovskite quantum dots (PQDs) with a larger size, and the results suggest that exciton confinement and defects likely play a more important role in these strongly quantum confined magic-sized clusters with a larger surface-to-volume ratio.

Perovskite quantum dots (PQDs) are a group of nanomaterials with perovskite ABX_3_ structure that, due to the quantum confinement effect (QCE), exhibit properties distinct from bulk perovskite.^1,2^ They are generally of interest in the context of their optoelectronic applications due to their attractive properties, including their size- and composition-tunable absorption and emission through the visible range; high photoluminescence quantum yield (PLQY) ranging from 50 to 90%; and low cost, facile synthesis.^2−5^

Recently, PQDs have garnered interest in spintronics applications due to their strong spin–orbit coupling (SOC).^6^ A strong SOC is desirable, because it facilitates spin selective optical manipulation and efficient spin generation and manipulation. Although strong SOC enables facile spin injection, a recent study has demonstrated that modulating the SOC by altering the PQD composition can increase the spin relaxation lifetime.^7−9^ This spin relaxation lifetime, *T*_1_, is known as the longitudinal spin relaxation time and is a typical metric for assessing the suitability of a material for spintronics and spin-based quantum information science applications. Despite the growing interest in the spin dynamics of PQDs and related systems, a study of perovskite nanocrystals with small size and strong quantum confinement has remained challenging due to their instability in comparison to larger perovskite structures.^10,11^

Perovskite nanoclusters, including magic-sized clusters (PMSCs) with single size or very narrow size distributions, are a highly quantumly confined perovskite nanocrystal with a size smaller than typical PQDs.^11,12^ Their strong quantum confinement is associated with significantly blue-shifted first excitonic absorption and emission bands. A typical methylammonium (MA) lead bromide (CH_3_NH_3_PbBr_3_) PMSC exhibits a sharp and narrow first excitonic absorption peaked at about 440 nm contrasted with the broad first excitonic absorption feature typically peaked around 520 nm associated with PQDs.^12−14^ In addition to their small size at approximately 2.8 nm, their absorption and emission bands with a small fwhm around 10 nm indicate a relatively monodispersed size distribution.^11,15^ PMSCs are considered an important stable intermediate in the development of PQDs and have recently attracted interest due to their properties which distinguish them from PQDs, such as an intrinsic chiral signal as measured by circular dichroism (CD) in the absence of chiral passivating ligands.^16−18^

The small size of PMSCs and correspondingly large surface-to-volume (S/V) ratio, with greater electron–hole confinement and often higher density of defects, are expected to lead to a shorter exciton lifetime in relation to PQDs.^15^ This expectation is consistent with a recent dynamics work where an exciton lifetime on the order of hundreds of picoseconds was measured in a PMSC with a low measured PLQY of 28%.^15^ However, it is not known if this reduction in exciton lifetime is persistent in high PLQY particles nor is it currently known how a highly confined size impacts the spin relaxation dynamics of MAPbBr_3_ PMSCs. To address this, this work employs circularly polarized transient absorption spectroscopy to measure the spin relaxation lifetime of ligand-passivated methylammonium lead bromide (MAPbBr_3_) magic sized clusters.

An adjusted hot ligand reprecipitation (HLARP) method was selected for PMSC synthesis. Previous work has shown that particles synthesized using the HLARP method exhibit superior stability and higher PLQY when compared to those synthesized at room temperature.^13^ Unlike past HLARP work, here a temperature controlled water bath and magnetic stirring were used alongside a strong acid (HBr) in PMSC precursor preparation. Femtosecond transient absorption (fs-TA) studies were conducted using both linear and circular pump polarizations, while time-resolved luminescence studies employed linear excitation. Full experimental details are available in the SI.

Figure 1 shows the ultraviolet–visible (UV) and photoluminescence (PL) spectra of MAPbBr_3_ PMSCs. The first excitonic absorption and corresponding emission are peaked at 447 and 454 nm, respectively, representing a significant blue shift compared to typical MAPbBr_3_ PQDs with first excitonic absorption and emission peaked at 512 and 521 nm, respectively, as shown in Figure S2.

**Figure 1:**
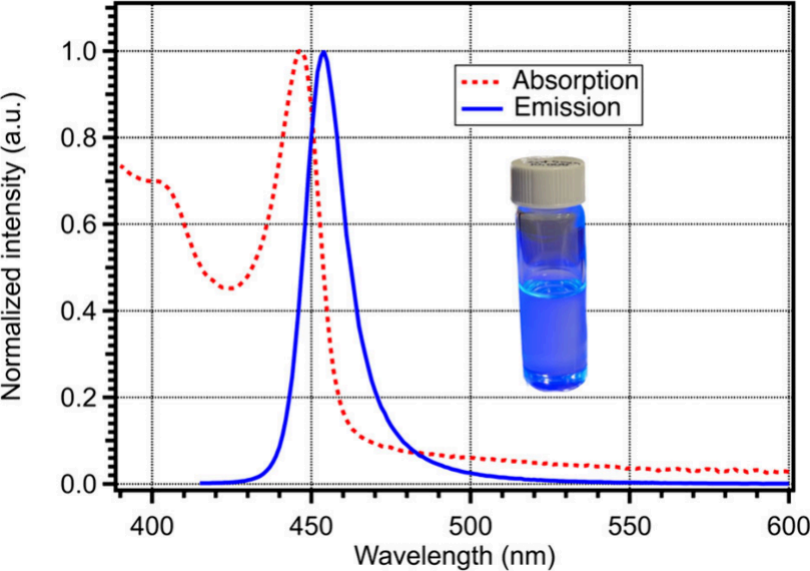
UV–vis (red dashed) and PL (blue solid) spectra of MAPbBr_3_ PMSCs. PL excitation was at 400 nm. Inset shows PMSCs in room light under irradiation by a 365 nm lamp.

Along with the peak position and small Stokes shift of 9 nm, the narrow PL profile with full width at half-maximum (fwhm) of 15 nm is characteristic of PMSCs and aligns with past literature.^12,13,15^ This narrow fwhm indicates that the population of nanocrystals is likely relatively monodispersed, consistent with a past study.^11,15^

Since nonlinear excitonic decay processes, such as Auger recombination, can occur on the same time scale as spin relaxation, a series of power dependent TA measurements were conducted.^19−21^ The power dependence study, shown in Figure S6, was conducted with pump powers ranging from 25 to 175 nJ. All measurements were taken by exciting above the band edge with 420 nm excitation and probing with a white light continuum in the UV region ranging from 400 to 500 nm. The selection of 420 nm excitation was to avoid the excitation of other states or species with absorption around 405 nm, as seen in Figure 1. The temporal delay between the pump and probe pulses ranged from −10 to 5000 ps to capture as much of the decay process as possible.

Along with the PIA peak at 458 nm, the spectra in Figure S6a show an additional absorption peak at around 430 nm. This absorption feature was not studied due to its proximity to spectral interference from the 420 nm excitation beam and the employment of a 435 nm long pass filter which interferes with the signal in this absorption region. Further fs-TA studies included in this work were conducted at powers well below the transition to the nonlinear power region.

Figure 2a shows the kinetic trace and fit from an fs-TA measurement with linear excitation extracted from the 448 nm bleach. This fs-TA measurement was performed with linear excitation to evaluate the overall exciton lifetime of the PMSCs. To gain better resolution on the slow component, which does not recover to the baseline in the 5 ns probed, TRPL was employed to better visualize the long lifetime component, as shown in Figure 2b.

**Figure 2:**
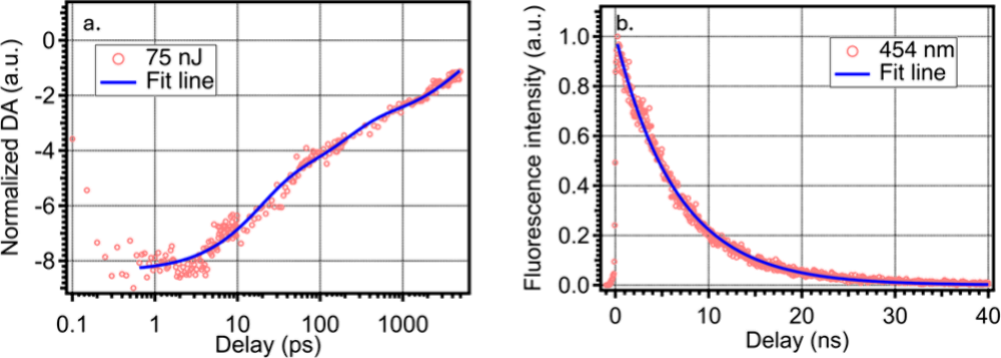
(a) Representative fs-TA kinetic trace and fit probed at the peak bleach magnitude at 448 nm with pump power at 75 nJ and (b) representative TRPL trace and fit with pump power at 25 nJ and probed at the peak photoluminescence magnitude at 454 nm.

The extracted kinetic trace probed at the 448 nm bleach shown in Figure 2a was fitted to a triple exponential fit function with time constants of 17 ps, 200 ps, and 5 ns. The TRPL trace shown in Figure 2b can be fit to a single exponential, with a lifetime of 6.5 ns. A multiple exponential recovery process in the bleach data indicates the presence of several competing forward and reverse processes and likely involves multiple states within the band gap.

Since the slow component of the bleach has a long lifetime that cannot be resolved over the total delay time, constrained by the delay stage of the laser system, the TRPL with longer time scale collection was used to better visualize the slow component. Thus, the single lifetime component of 6.5 ns observed in TRPL is attributed to the same recovery process as the slow component of the fs-TA bleach feature. This nanosecond lifetime is consistent with a high PLQY of 75% and indicates that the adaptations made to the PMSC synthesis have produced particles in which the exciton dynamics of the PMSCs are not dominated by defect-generated trap states.

Similarly, representative PQDs with a PLQY of 65% were studied using fs-TA and TRPL for comparison. For fs-TA with a linearly polarized pump, an excitation wavelength of 470 nm was used, with probe wavelengths from 500 to 750 nm. The fs-TA results are shown in Figure S6. The kinetic profile of the photoinduced bleach, centered at 512 nm, can be best fit to a triple exponential function with time constants of 41 ps, 400 ps, and 8.6 ns, and the fitting parameters are summarized in Table S4. The TRPL profile monitored at 520 nm was fit to a double exponential function with time constants of 400 ps and 8.6 ns. The overall or average carrier lifetime of the PQDs is longer than that of PMSCs, possibly due to stronger confinement and higher density of defects due to the larger surface-to-volume ratio of the PMSCs.

Figure 3 shows the fs-TA spectra acquired by using circularly polarized pump pulses and linearly polarized probe pulses for PMSCs. Figure 3a shows the cocircular pump and probe (σ^+^σ^+^), while Figure 3b shows the counter circular pump and probe (σ^+^σ^–^). Both Figure 3a and b were acquired with the left circularly polarized pump (σ^+^). Similarly, Figure 3c and d were acquired by the right (σ^–^) circularly polarized pump and show a co- (σ^–^σ^–^) and counter- (σ^–^σ^+^) circularly polarized pump and probe in c and d, respectively. Comparisons of these spectra show clear differences in the development of the PIA feature. These differences can be attributed to the preferential excitation of one electron spin population over another and is typical in circularly polarized transient absorption spectroscopy. Past work on PQD spin lifetime has produced pump–probe spectra with similar phenomena, although these PMSC spectra are distinct in several ways.

**Figure 3:**
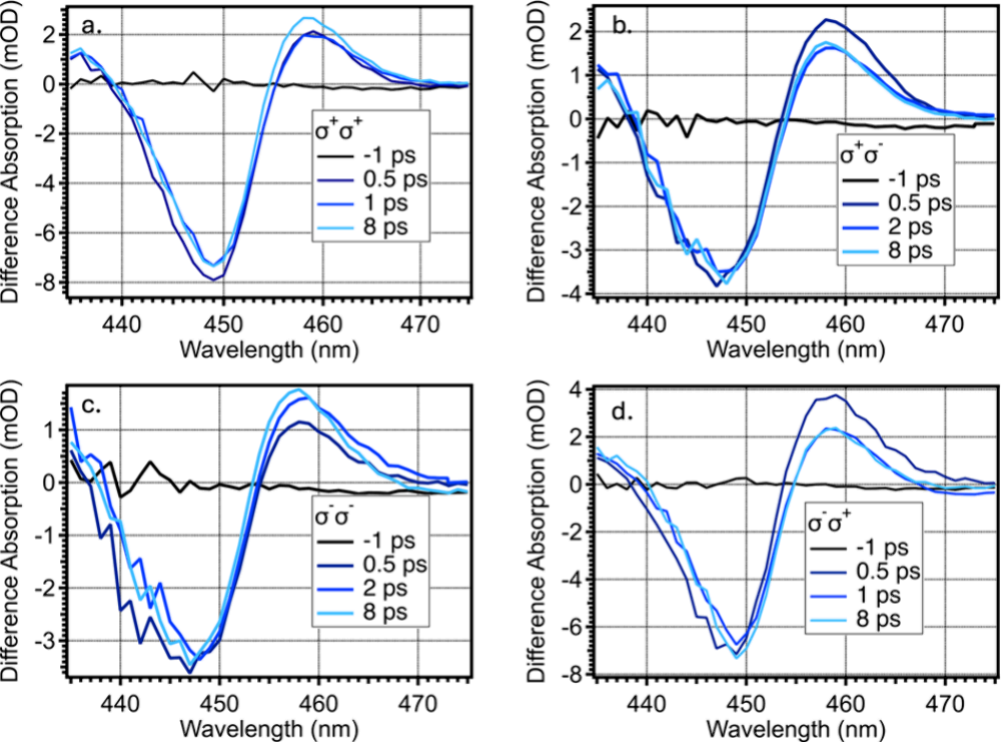
(a) Co-circular (σ^+^σ^+^) and (b) counter-circular (σ^+^σ^–^) fs-TA PMSC spectra with excitation power of 25 nJ and ranged probe times from −1 to 8 ps.

First, consistent with the pump–probe spectrum seen in Figure S2, the PIA feature persists throughout the TA measurement, in contrast to PQDs which typically exhibit a short-lived PIA feature dominated by bleach kinetics at later time points. Second, as observed in previous PQD dynamics studies, the PIA feature is typically observed only with a counter-circular pump and probe and is absent with a cocircular pump and probe.^7,8^ However, in the case of the PMSCs here, the PIA feature persists regardless of the circularity of the pump and probe and is observable in any combination of circularity. However, there are key differences in the PIA rate of growth when comparing the co- and counter-circular pump and probe. Specifically, the PIA feature in the cocircular pump and probe spectrum (Figure 3a) reaches its maximum intensity after about 8 ps, indicating slower growth in intensity compared to the counter-circular pump and probe spectrum (Figure 3b), which reaches peak intensity within 0.5 ps, rapidly decays over the following 8 ps, and then equalizes with the cocircular pump and probe spectrum dynamics. The distinct dynamics suggested by the pump probe spectra are consistent with established theory regarding spin-selective excitation, where left- and right circularly polarized excitation preferentially populates different states in the conduction band.^6^ This is shown in Figure S9 in the comparison of left and right circular pumps. Although the precise conduction band (CB) and valence band (VB) J states are unknown for the PMSC system, Figure S9 and accompanying discussion qualitatively depict state selection using left or right circularly polarized light.

The disparate dynamics observed in the PMSCs fs-TA spectra in Figure 3 are better illustrated by extracting their associated kinetic traces, shown in Figure 4. Figure 4a shows the co- (σ^+^σ^+^) and counter-circular pump and probe spectra (σ^+^σ^–^) normalized to their corresponding intensities at 200 ps for the PMSCs. This normalization allows for the evaluation of the kinetic differences that occur at early time points, which are associated with the spin-selective excitation and subsequent spin-relaxation process. These kinetic traces were obtained using a left circularly polarized pump and are contrasted against similar traces obtained with right circularly polarized pump shown in Figure 4b. For the PMSCs, the co- (σ^–^σ^–^) and counter- (σ^–^σ^+^) circular pump and probe traces are inverted in relation to Figure 4a. Left- and right-circularly polarized pump pulses carry spin angular momentum with opposite signs. Therefore, inversion in the observed kinetics is expected when comparing the two circular pump polarities and indicates that they are dominantly filling different states probed.^6^ Similar inversion behavior is observed in Figure 4c and d for the PQDs when switching between left- and right-circularly polarized excitation (pump) light. Further, it has been demonstrated that the spin relaxation lifetime can be extracted from the kinetic traces by simple subtraction of the two over the probe range.^6−9^ These extracted data are shown in Figure 4a and b for the PMSCs and in Figure 4c and d for the PQDs. In each case, the subsequent data are fit to a single exponential fit function convolved with a Gaussian instrument response function (IRF).

**Figure 4:**
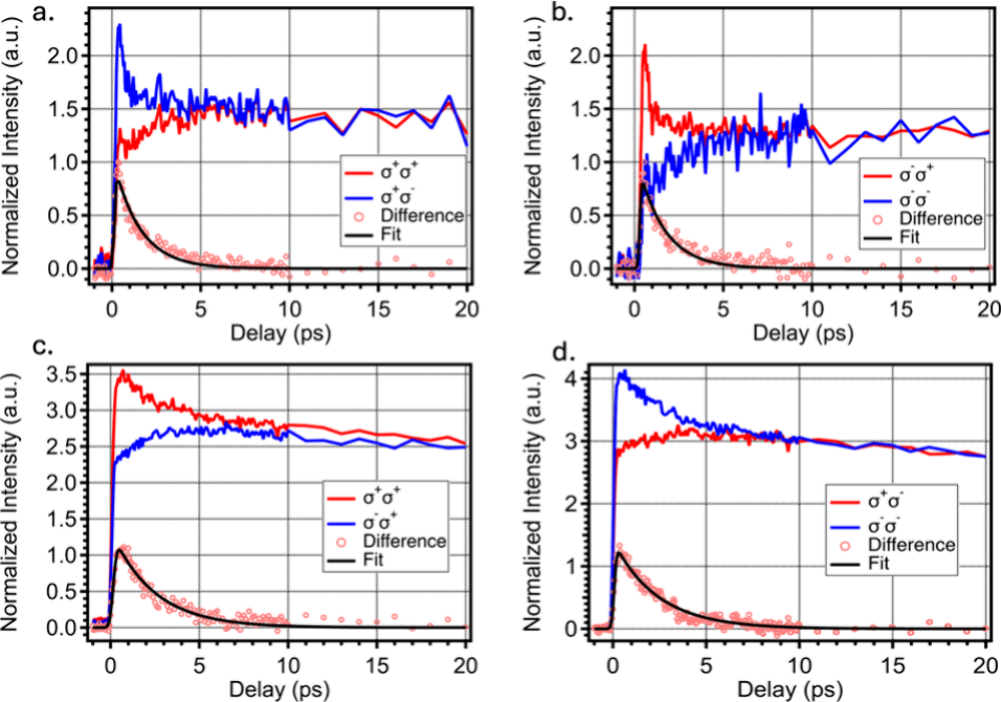
(a, b) Extracted kinetic traces from co- and counter-circularly polarized spectra for PMSCs and (c, d) extracted kinetic traces from co- and counter-circularly polarized spectra for PQDs. The difference between the co- and counter-circular pump–probe spectra is plotted and fit to a single exponential.

For the PMSCs, the extracted decay profile is best fit with a single time constant of 1.5 ps, while the PQDs are best fit to a time constant of 2.4 ps. In each case, since the experimental measurements cannot distinguish between electron or hole relaxation, these relaxation lifetimes are best considered as average values of electron and hole spin. With the assumption that the electron–hole pair is bound, they practically reflect the spin relaxation time of the exciton.

Spin relaxation involves several different mechanisms. The Elliot–Yafet (EY) mechanism, commonly attributed as the dominant spin relaxation mechanism at room temperature for systems with spatial inversion symmetry or strong spin polarization, is based on spin-flip electron–phonon or electron-impurity scattering.^9,22−24^ In addition to EY, common mechanisms in quantum dots include D’yakonov–Perel (DP) for materials with broken inversion symmetry and electron hole exchange (EHE).^25−30^ It is expected based on past experimental and theoretical studies that electron–phonon interactions in nanostructures are reduced in comparison to bulk due to a reduced density of states.^8,9,31,32^ Conversely, when electron–phonon interactions are reduced, the EHE mechanism is expected to increase in dominance, especially in systems with strong Coulombic attraction within the photoexcited exciton due to stronger spatial confinement.^32,33^

Since the PMSCs are strongly quantum confined spatially, the EY spin-flip electron–phonon scattering mechanism is expected to be heavily suppressed, while DP and EHE mechanisms are expected to become more dominant.^8,31^ In MAPbX_3_ perovskite bulk, the orthorhombic phase has been associated with large Rashba splitting, associated with broken inversion symmetry, and further, in MAPbBr_3_, the intrinsic CD signal has been associated with the orthorhombic phase.^34,35^ In PMSCs, both an orthorhombic phase and intrinsic chirality as determined from circular dichroism studies have been reported previously.^13,16^ Therefore, since broken inversion symmetry is suggested in PMSCs, it is likely that the DP mechanism is more dominant in spin relaxation in relation to the EY.

In summary, the spin relaxation lifetime of excitons in strongly confined MAPbBr_3_ PMSCs was measured for the first time. The measured 1.5 ps spin lifetime, shortened in relation to larger MAPbBr_3_ PQDs, measured at 2.4 ps, highlights the importance of nanocrystal size on the spin relaxation time. Strong confinement and increased density of defects seem to outweigh the effect of electron–phonon interaction in spin relaxation, resulting in the increasing dominance of alternative mechanisms like D’yakonov–Perel and electron hole exchange.

## Supporting Information Available

Experimental details, power dependent TA, wavelength and power dependent TRPL, additional discussion (PDF)
