# Supplementary material for: Ultrafast Spin Relaxation of Charge Carriers in Strongly Quantum Confined Methylammonium Lead Bromide Perovskite Magic-Sized Clusters
Source: ACS Phys Chem Au. 2024 Sep 16;4(6):610–4. doi: 10.1021/acsphyschemau.4c00051 (PMC11613346; doi:10.1021/acsphyschemau.4c00051)
Supplement: Supplementary file 1 — pg4c00051_si_001.pdf [file pg4c00051_si_001.pdf]

Supporting Information

Ultrafast Spin Relaxation of Charge Carriers in Strongly Quantum Confined Methylammonium Lead Bromide Perovskite Magic-Sized Clusters

*David C. Zeitz[1], Vivien L. Cherrette[1], Sarah A. Creech[1], Yan Li[2], Yuan Ping[3], and Jin Z. Zhang[1]**

[1]*Department of Chemistry and Biochemistry, University of California, Santa Cruz, CA 95064, USA*

[2]*School of Materials Science and Engineering, University of Science and Technology Beijing, Beijing 100083, P.R. China*

3Department of Materials Science & Engineering, University of Wisconsin, Madison, WI 53706 USA

*Corresponding Author. Email: zhang@ucsc.edu

Experimental Methods

Materials

Methylammonium bromide (MABr, 98%, Sigma-Aldrich), $PbBr_2$ (98+%, Thermo Scientific), oleic acid (analytical grade, Fisher), oleylamine (70% Tech Grade, Sigma-Aldrich), N,N-dimethylformamide (DMF, Oakwood Chemical), toluene (99.9%, Sigma-Aldrich), and hydrobromic acid (HBr, Honeywell, 48%). All chemicals were used as received without any further purification.

Hot Ligand Assisted Reprecipitation (HLARP) Synthesis of $MAPbBr_3$ PMSCs

Methylammonium lead bromide ($CH_3NH_3PbBr_3$) PMSCs were synthesized using hot ligand assisted reprecipitation (HLARP). 0.080 mmol (9.0 mg) of MABr and 0.080 mmol (29.0 mg) of $PbBr_2$ were dissolved in 400 μL DMF with the assistance of 20 μL concentrated HBr in a glass vial. The solution was heated in a water bath set at 70˚C with magnetic stirring set at 1150 rpm for two minutes until dissolved. 65 μL of oleic acid (OA) was added to the mixture and stirred at 70˚C for two minutes. The precursor transitioned from colorless to a clear, golden yellow solution. Then, 75 μL oleylamine (OAm) was added to the mixture and heated at 70˚C for an additional two minutes. 30 μL of the resultant precursor was swiftly injected into 5 mL room temperature toluene under vigorous stirring at 1150 rpm. To remove larger particles and aggregates the sample was centrifuged at 10000 rpm for five minutes and the supernatant was retained. The colloidal PMSCs were allowed to mature at room temperature in ambient conditions overnight before further study.

Synthesis of $MAPbBr_3$ PQDs

Methylammonium lead bromide PQDs were synthesized by injection of room temperature precursor solution into 4.20 mL of rapidly stirring toluene at 70°C. The precursor solution was prepared by dissolving 0.10 mmol MABr (11.0 mg) and $PbBr_2$ (36.0 mg) in 1.00 mL of DMF by ultrasonication for 5 minutes. 200 μL of OA and 18 μL OAm were added to the solution and sonicated for an additional 3 minutes. 140 μL of the resultant precursor solution was injected into the rapidly stirring hot toluene. A turbid yellow product with green luminescence was immediately formed, and the product was removed from heat immediately following injection and allowed to cool to room temperature naturally. Last, to remove larger particles and aggregates, the product

was centrifuged at 5000 rpm for five minutes, and the clear, yellow-green supernatant was retained for characterization.

Ultraviolet−visible (UV-Vis) and photoluminescence (PL) spectroscopy

UV−Vis absorption spectra of the colloidal samples were measured with an Agilent Technologies Cary 60 UV−Vis. The PL was measured using an Agilent Technologies Cary Eclipse Fluorescence Spectrophotometer.

Photoluminescence Quantum Yield measurement

PLQY was measured against a quinine sulfate in 0.1 M sulfuric acid standard using the method established by Yvon et al. The PLQY was calculated using Equation S1.

$$\Phi_X = \Phi_{ST}\left(\frac{Grad_X}{Grad_{ST}}\right)\left(\frac{\eta_X{}^2}{\eta_{ST}{}^2}\right) \qquad \text{(Eq. S1)}$$

Transient Absorption Spectroscopy

Femtosecond TA measurements were taken using a Light Conversion laser system. The fundamental beam with an output fundamental frequency of 1030 nm, 10 W power, a repetition rate of 20 kHz, and pulse width of 160 fs was generated by PHAROS (Light Conversion). PHAROS is equipped with a ytterbium-doped potassium gadolinium tungstate (Yb:KGW) lasing medium. Tunable pump wavelengths were produced by feeding 80% of the fundamental beam into an optical parametric amplifier (OPA), ORPHEUS (Light Conversion). A white light continuum probe beam was generated by passing the remaining 20% of the fundamental through a nonlinear sapphire crystal. The generated pump and probe beams were aligned in an ultrafast TA system, HARPIA (Light Conversion). Spatial alignment was controlled by mirrors, while temporal overlap was controlled by a translating delay stage. For measurements, the time delay

between pump and probe pulses was ranged from -10 to 5000 ps. Data was collected using the Light Conversion HARPIA data acquisition software and using an Oxford Instruments Andor spectrometer.

Polarization-dependent Transient Absorption Spectroscopy

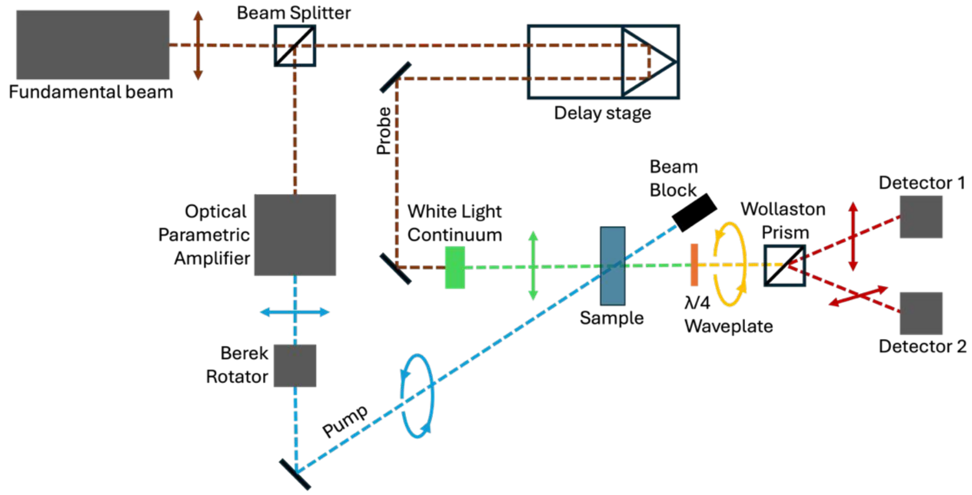

Figure S1 Laser layout for circularly polarized pump-probe experiments. Linear beam is indicated by a straight double ended arrow. Circularly polarized beam is indicated by round arrow. Pump circularity can be selected for either left or right circular polarization.

Transient absorption measurements were carried out using the femtosecond laser system discussed above, but with several further inclusions. Since the fundamental output is horizontally linearly polarized, it was directed through a Berek rotator. This motorized rotator allowed for the selection of crystal rotation and tilt angles that corresponded to either left- or right-circular polarization. This generated the circularly polarized pump beam.

The probe beam, also horizontally linearly polarized from the fundamental, was allowed to pass through the sample as normal. Subsequently, it was converted to circular polarization using

a λ/4 waveplate. A Wollaston prism was used to split the horizontal and vertical components of the circular probe. Finally, the two probe signals were measured individually but simultaneously using a pair of vis-array detectors mounted to an Oxford spectrometer.

A Glan-Taylor polarizer was used to assess pump circularity while probe circularity was verified using an automated polarizer paired with a photodiode. Data was collected using the Light Conversion Dual Detection software and using an Oxford Instruments Andor spectrometer.

Results

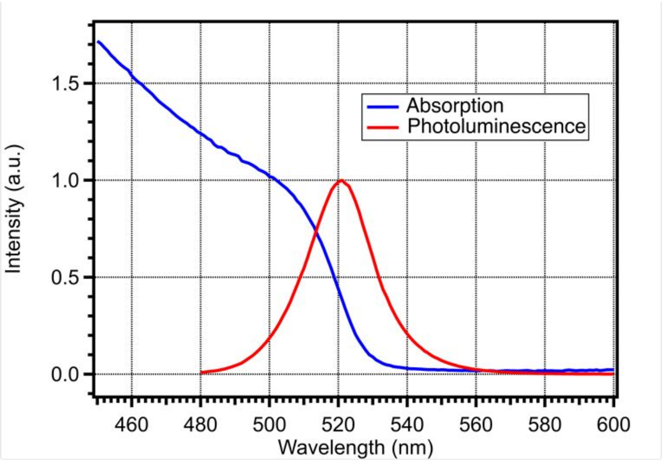

Figure S2 UV-Vis (blue) and PL (red) spectra of $MAPbBr_3$ PQDs. PL excitation was at 470 nm.

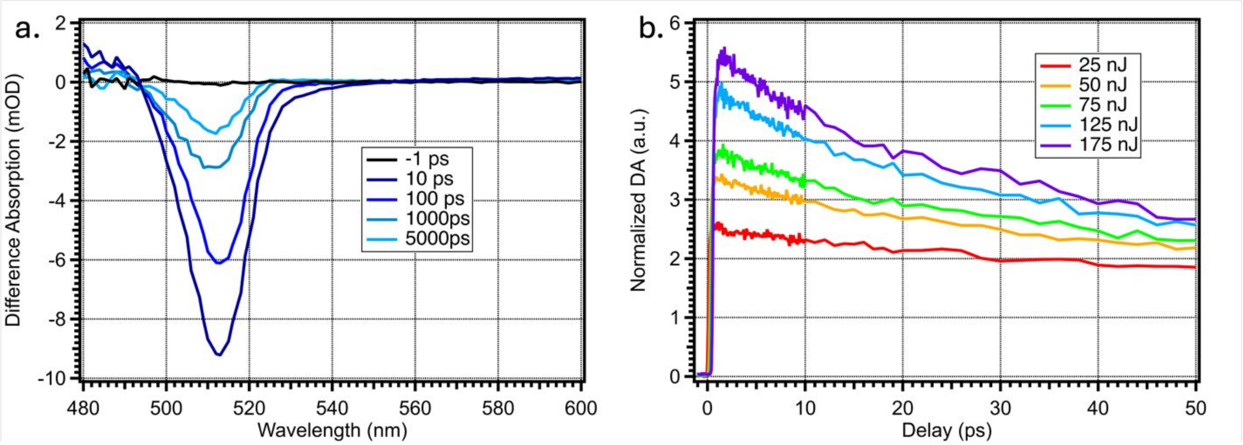

Figure S3 (a) Representative PQD pump-probe spectrum at pump power of 50 nJ and (b) power dependent dynamics of bleach.

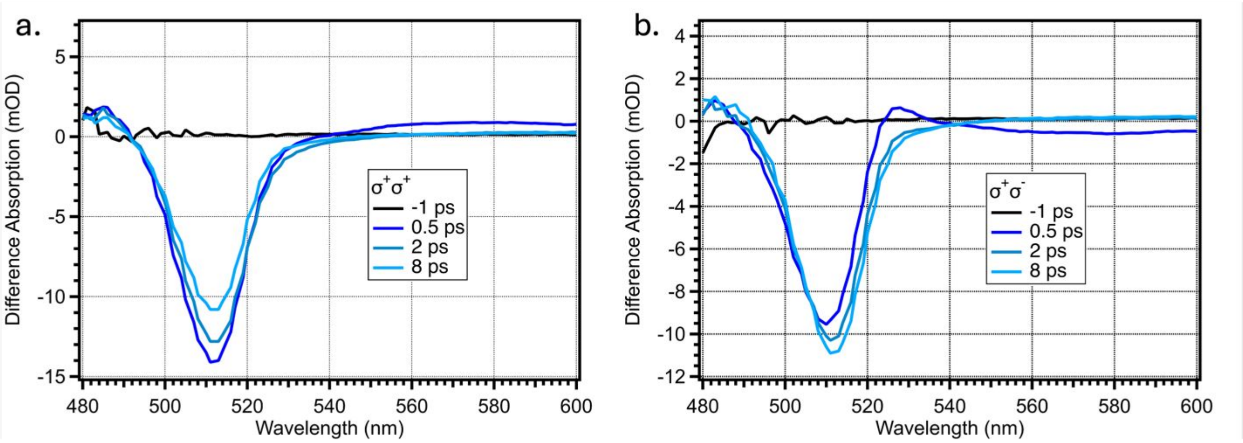

Figure S4 (a) Co-circular ($\sigma^+ \sigma^+$) and (b) counter-circular ($\sigma^+ \sigma^-$) fs-TA PQD spectra with excitation power of 25 nJ and ranged probe times from -1 to 8 ps.

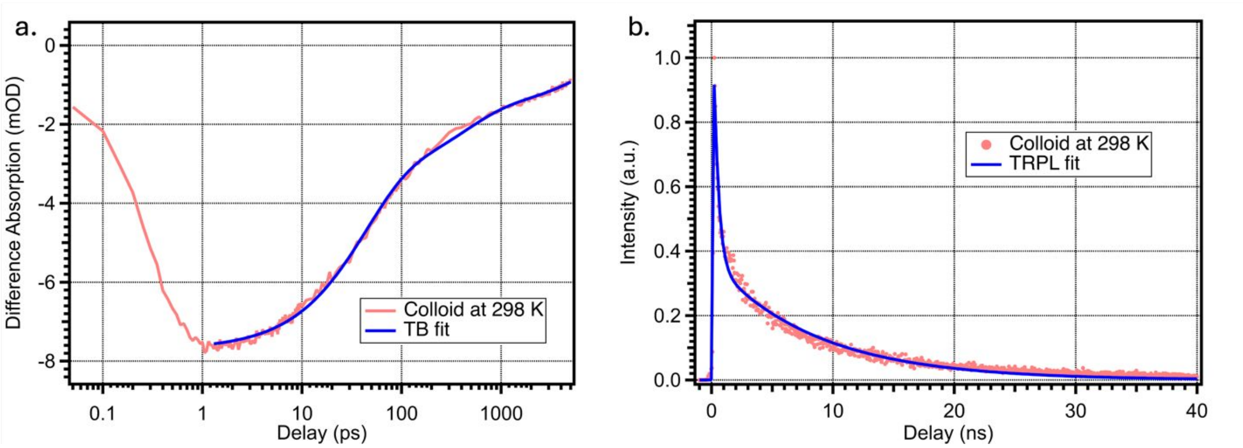

Figure S5 (a) representative TA kinetic trace and fit probed at peak bleach magnitude at 512 nm and (b) representative TRPL trace and fit with pump power at 25 nJ and probed at the peak photoluminescence magnitude at 520 nm.

Power dependent TA Results of PMSCs

Two key TA features were probed for power dependence, the bleach feature peaked at 448 and the PIA feature peaked at 458 nm, which can be seen in the representative pump-probe spectrum shown in Figure S1a. Figure S1b shows the extracted kinetic traces from these features, with the PIA in the main panel and the bleach in the inset. The PIA traces were normalized to their peaks to assess the addition of an additional fast process at early time scales. Similarly, the bleach traces were normalized to their minimum signal magnitude at 5 ns to visualize the signal growth with increasing pump intensity at early time points.

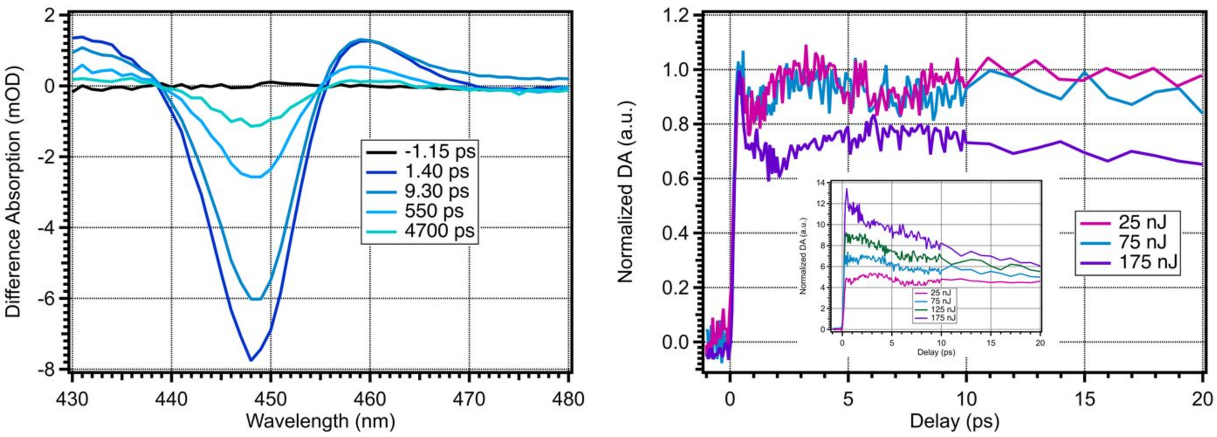

Figure S6 (a) Representative PMSC pump-probe spectrum at pump power of 75 nJ and (b) power dependent dynamics of PIA (main panel) and bleach (inset).

This study shows that non-linear effects are not involved in the exciton dynamics at powers at or below 125 nJ.

Similarly, both power and wavelength dependent TRPL measurements were taken to ensure there were no power dependent impacts on the observed fluorescence lifetime.

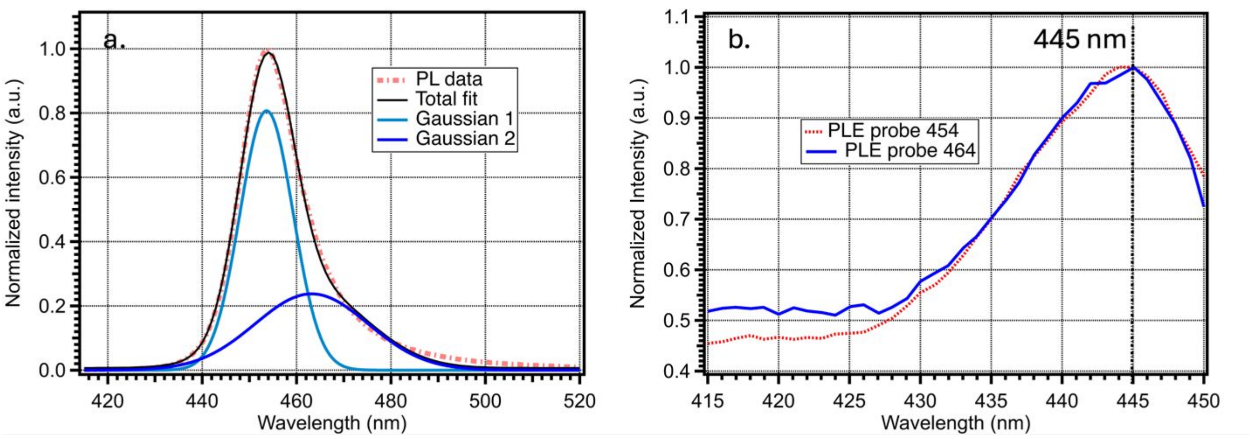

Figure S7 (a) Deconvolved double Gaussian fit to photoluminescence emission spectrum, and (b) PLE spectra probed at each Gaussian peak.

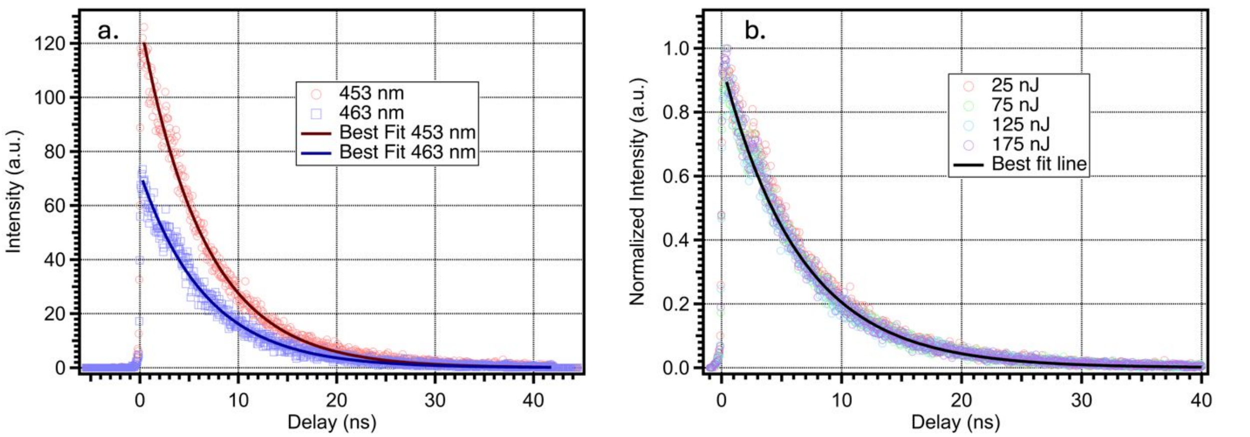

Figure S8 Wavelength (a) and power (b) dependent TRPL.

Both the wavelength and power dependent TRPL show consistent fits to ~6.5 ns as detailed in Table S3 below.

The radiative and nonradiative lifetimes were calculated using the following equations.

$$\tau_r = \frac{\tau_{obs}}{PLQY} \qquad \text{(Eq. S2)}$$

$$\frac{1}{\tau_{obs}} = \frac{1}{\tau_r} + \frac{1}{\tau_{nr}} \qquad \text{(Eq. S3)}$$

Here, the radiative lifetime (τ_r) was calculated using the PLQY. τ_r, along with the observed lifetime (τ_{obs}) of 6.5 ns from the TRPL decay curve was used to calculate the nonradiative lifetime (τ_{nr}). It was found that τ_{nr} has a lifetime of about 27 ns, three times longer than that of τ_r, calculated at about 8.7 ns. This indicates that the PMSC exciton recombination is primarily radiative, suggesting diminished trap state influence on the overall dynamics. Since spin relaxation occurs on a time scale faster than each of these individual processes and exciton recombination overall, the TA dynamics with linear excitation are not subject to further modelling.

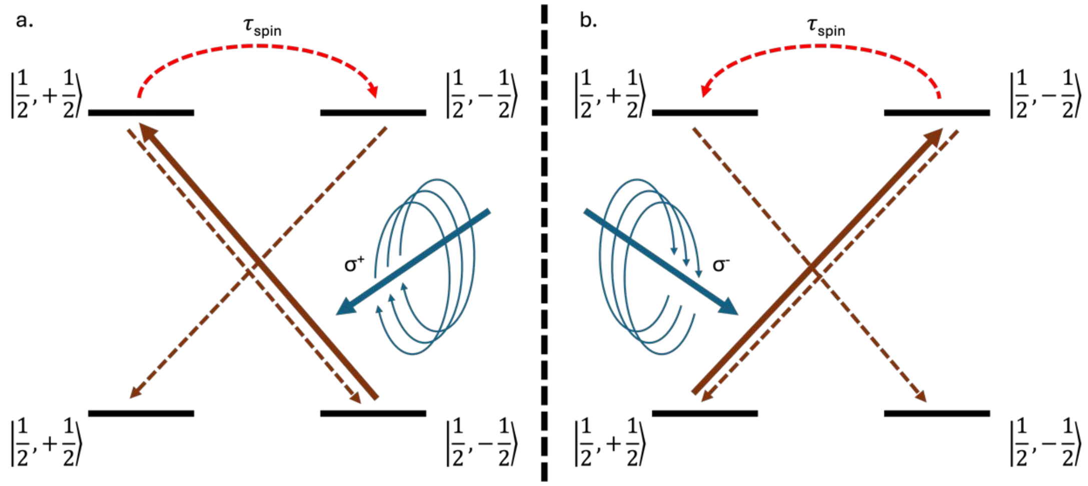

Figure S9 Graphic depicting spin selective excitation from the valence to conduction band with left (a) and right (b) circularly polarized light. Solid lines show absorption and dashed lines show spin relaxation and subsequent recombination.

We note that the exact J and mJ values are practically unknown given the exact chemical composition of MSC remains to be determined; here is a qualitative discussion how photon carries angular momentum and selects spin by light helicity. In the case of left pump (Figure S5a), the dominantly filled state corresponds to electron excitation from the valence band (VB) $\left|\frac{1}{2}, -\frac{1}{2}\right\rangle$ to

the conduction band (CB) $\left|\frac{1}{2}, +\frac{1}{2}\right\rangle$. The CB $\left|\frac{1}{2}, -\frac{1}{2}\right\rangle$ state is not preferentially populated, consistent with the σ^+ momentum of +1. This is inverted in the case of right pump (Figure S5b), where right circular pump preferentially excites electrons from the (VB) $\left|\frac{1}{2}, +\frac{1}{2}\right\rangle$ to the CB $\left|\frac{1}{2}, -\frac{1}{2}\right\rangle$. Correspondingly, the CB $\left|\frac{1}{2}, +\frac{1}{2}\right\rangle$ state is not preferentially filled by σ^- excitation.

Fitting results

Data were fit to exponential fit functions of the form

f(x) = y0+(A*EXP(-x/t1))+(B*EXP(-x/t2))+…

Where the y-axis offset (y0) was forced to a zero baseline.

Table S1 Fitting parameters of PMSC TA signal probed at 448 nm at power of 75 nJ.

y0	A (mOD)	t1 (ps)	B (mOD)	t2 (ps)	C (mOD)	t3 (ps)
0 (held)	-3±1	17±5	-2.1±0.6	200±50	-2.9±0.9	5000±1000

Table S2 Fitting parameters of PMSC TRPL signal monitored at 454 nm at power of 25 nJ.

y0	A (a.u.)	t1 (ns)
0 (held)	1.0±0.1	6.5±0.6

Table S3 Fitting parameters of PMSC TRPL signal monitored at 453 nm and 463 nm at power of 25 nJ.

	y0	A (a.u.)	t1 (ns)
453 nm	0 (held)	125±13	6.5±0.6

463 nm	0 (held)	72±7	6.5±0.6

Table S4 Fitting parameters of PQD TA signal probed at 510 nm.

y0	A (mOD)	t1 (ps)	B (mOD)	t2 (ps)	C (mOD)	t3 (ns)
0 (held)	-4.3±0.4	41±4	-1.8±0.2	400±50	-1.7±0.9	8.6±0.9
